# Can supervision promote domestic waste sorting? Evidence from China

**DOI:** 10.3389/fpubh.2026.1895977

**Published:** 2026-07-29

**Authors:** Qian Li, Meifang Zhou

**Affiliations:** College of Economics, Beijing Technology and Business University, Beijing, China

**Keywords:** community governance, environmental supervision, policy intervention, public health, waste sorting

## Abstract

Proper waste sorting is a fundamental aspect of constructing an ecologically sustainable civilization and a vital step towards enhancing urban living standards and public health. Using survey data from urban residents in Beijing, this paper empirically examines the impact of waste sorting supervision on residents’ domestic waste sorting. The impact is tested through the multiple mediators model to accommodate three potential mediating mechanisms, which are the awareness of waste sorting significance, standards and policies. The results show that despite 82.1% of urban residents showing varying degrees of participation in domestic waste sorting, there is still considerable room for improvement in promoting it. The implementation of waste sorting supervision appears to be an effective strategy to drive urban residents’ involvement in domestic waste sorting. However, the effect of such supervision on residents’ waste sorting exhibits marked heterogeneity in age and income, with a more pronounced effect among low-income and younger residents relative to their high-income and older counterparts. In terms of the mediating mechanism, waste sorting supervision promotes residents’ involvement in domestic waste sorting by improving their awareness of the significance, standards, and policies of domestic waste sorting.

## 1 Introduction

The acceleration of urbanization and the surge in urban residents’ consumption have led to a substantial increase in the generation of domestic waste in urban areas. Inadequate disposal of excessive domestic waste particularly when unsorted can inflict adverse repercussions on both ecological integrity and public health through multiple pathways, including leachate contamination of water sources, toxic air emissions from open burning, and increased transmission of vector-borne diseases (1, 2). These health externalities underscore the necessity of improving residents’ sorting behavior at the source. Domestic waste sorting is a globally endorsed practice that can reduce the volume of waste generated and enhance the efficacy of waste processing. Many countries and regions have been proactively encouraging or mandating domestic waste sorting among their residents. For example, Sweden has applied policy instruments such as deposit system, waste fee and extended producer responsibility (3), while Danish has outlined a target of recycling 50% of total household waste by the year 2022 and transitioning to a circular economy in the future (4). China, as one of the world’s most populous countries, has instituted domestic waste sorting as a policy for over two decades and introduced a range of laws and regulations to guide and facilitate residents’ participation. Shanghai implemented the first local urban regulation in China, *Shanghai Household Waste Management Regulation* on July 1, 2019, which led China into a new era of waste sorting (5, 6). However, the persistent problem of mixed collection and transportation in many cities, coupled with insufficient incineration capacity, has to some extent undermined residents’ confidence in front-end sorting efforts (6).

The implementation of laws and regulations pertaining to municipal waste sorting signifies progressively stringent standards and more rigorous supervision requirements. Waste sorting management with third-party supervision is a new mode for many cities in developing countries (7). Some Chinese communities have designated garbage can watchers, who are typically older adults individuals employed or those volunteering for the position. Their principal duties include maintaining sanitation standards at garbage disposal points, providing guidance to residents on the appropriate disposal of domestic waste, facilitating the work of garbage collectors, and supervising resident compliance with waste sorting policies. Also, these attendants play a role in raising public awareness about the importance of domestic waste sorting within their communities. While such supervision may suggest a compulsory approach, it remains to be determined whether these regulations have indeed increased residents’ participation in domestic waste sorting.

Environmental supervision at the macro level has been found to produce a range of outcomes, including industrial structural optimization (1), industrial green and low-carbon transformation (8), and regional green transformation (9), among others, and to positively impact economic development. At the micro level, environmental supervision can encourage residents’ pro-environmental behaviors, including manure recycling (10) and fertilizer application reduction (11). In contrast to macro-level environmental supervision and other residents’ behaviors, the supervision of domestic waste sorting at the micro level is distinct in several respects: firstly, due to the decentralized and irregular scheduling of domestic waste delivery, it is difficult for local governments to implement all-round and effective supervision on the waste sorting activities of residents (7); secondly, the regulation of domestic waste sorting is designed primarily to raise awareness rather than to impose punitive measures; and thirdly, the third-party supervision mode is mainly adopted as the workload has exceeded the capacity of government departments (7). Given these unique characteristics, examining the effect of waste sorting supervision on residents’ behavior can serve as a valuable complement to existing research.

A growing body of literature has empirically examined the relationship between supervision (or policy enforcement) and residents’ waste sorting behavior, with most studies confirming a positive and significant effect. Nevertheless, significant gaps between regulatory ambitions and operational outcomes remain (12). And the effectiveness dependes not only on policy design, but also on the sociocultural conditions into which these instruments are introduced (13). It also requires the coordinated cooperation of multiple stakeholders or instruments (14, 15). For instance, Chen et al. (16) found that compulsory source-separated policies significantly improved household waste sorting performance. Liu et al. (1) reported that public environmental supervision promotes residents’ pro-environmental behaviors, including waste sorting. Beyond direct policy effects, researchers have extensively employed the Theory of Planned Behavior (TPB) or extended TPB to understand the underlying mechanisms. Several studies have demonstrated that subjective norms which reflect perceived social pressure, often shaped by supervision and community oversight—are a key predictor of waste sorting intention and behavior (17–22). Govindan et al. (23) built an extended TPB model and found that residents’ waste sorting intentions were positively and significantly correlated with their attitudes, subjective norms, and perceived behavioral control. Zhang et al. (19) further confirmed that subjective norms play a critical role in transforming intention into actual sorting behavior, and Ao et al. (20) identified subjective norms as a major driving factor among rural residents. Recent intervention studies have provided further direct evidence: a field experiment in the Yangtze Delta region showed that after a 1-month supervision period, the source separation rate of waste bins dramatically rose from 13 to 90%, whereas education and communication alone did not yield comparable improvements (24). Similarly, a study in Shanghai found that assistance and supervision positively moderated the relationship between sorting intention and actual behavior, suggesting that supervision helps bridge the intention-behavior gap (25). In terms of mechanisms, prior research has identified awareness and knowledge as important mediators. Ma et al. (26) demonstrated that policy instruments affect waste separation behavior through the mediation of perceived value. Tian et al. (27) showed that policy implementation increases public waste sorting behavior by improving policy awareness and knowledge, and supervision can enhance residents’ understanding of sorting standards and significance (28). These studies suggest that supervision works not only through deterrence but also through cognitive pathways, aligning with our proposed mediating hypotheses.

In view of this, the present paper uses survey data from urban residents in Beijing to empirically examine the effect of domestic waste sorting supervision on residents’ sorting behavior. The possible marginal contributions are threefold: First, while previous studies have largely relied on data from pilot cities such as Shanghai and Nanjing, empirical evidence from Beijing—the capital city of China, which possesses a distinct political status, diverse demographic composition, and unique policy implementation environment—remains scarce. By utilizing first-hand survey data from Beijing residents, our study provides a contextually valuable perspective that complements and extends the existing literature. Second, although the existing literature has confirmed the positive role of supervision, few studies have systematically investigated the mediating mechanisms through which supervision influences behavior particularly the roles of awareness of significance, standards and policies, and our study aims to fill this gap. Third, considering the age and income heterogeneity in the effect of waste sorting supervision on residents’ waste sorting behavior, our findings can carry more practical significance for targeted policy design.

The rest of the paper proceeds as follows: Section 2 presents a theoretical analysis and research hypotheses; Section 3 describes the methodology, followed by Section 4 detailing the analysis of empirical results; and lastly, the conclusion section provides a summary of findings, policy implications and limitations.

## 2 Theoretical framework

As waste sorting supervision primarily pertains to communities, this study develops a dynamic game analysis framework consisting of two subjects—the communities and the residents—based on the approach put forward by Li et al. (29). We present this model as an auxiliary theoretical illustration to clarify the strategic logic underlying the interaction between communities and residents, and to derive the directional predictions that guide our empirical analysis. The model is not intended for direct parameter estimation or empirical testing, as its structural parameters were not measured in our questionnaire.

The framework assumes complete information exchange between the community and the residents. This simplifying assumption is adopted to facilitate analytical tractability and to clearly illustrate the core strategic logic of the interaction. We acknowledge that in reality, information asymmetry between communities and residents is common. Residents may not fully perceive the actual intensity of supervision or the probability of penalties, while communities cannot perfectly observe residents’ waste sorting behavior. However, for the purpose of deriving the basic directional predictions, the complete information assumption suffices.

For communities, it is assumed that their cost of implementing domestic waste sorting supervision is $C_{1}$ (all parameters are assumed to be non-negative in this study), and the fine *F* is levied on residents for failing to sort their domestic waste. Given the complexity of implementing the supervision on domestic waste sorting, it is difficult to achieve a one-hundred-percent regulation (30). This study assumes that the probability of a resident being caught by a supervisor and penalized for failing to sort waste is denoted as τ . With respect to residents, it is assumed that the loss of reputation resulting from their failure to sort domestic waste and being detected by the supervisor is $R_{1}$ , the cost of sorting domestic waste is $C_{2}$ , and the benefit generated from waste sorting is $R_{2}$ . It is also assumed in the study that the probability of the community engaging in waste sorting supervision is $q_{1}$ , while the probability of non-supervision is $\left(1-q_{1}\right)$ . Additionally, the probability of residents participating in domestic waste sorting is $p_{1}$ , whereas the probability of non-participation is denoted as $\left(1-p_{1}\right)$ . It should be noted that $p_{1}$ and $q_{1}$ fall between 0 and 1.

Building upon the aforementioned assumptions, the expected utilities of the residents ( $U_{\text{resident}}$ ) and the community ( $U_{\text{community}}$ ) can be expressed into the following Equations 1, 2 respectively:


$$\begin{array}{l} U_{\text{resident}}=p_{1}[q_{1}(-C_{2}+R_{2})+(1-q_{1})(-C_{2}+R_{2})]\\ +(1-p_{1})q_{1}(-\tau R_{1}-\mathrm{\tau F}) \end{array}$$ (1)

$$U_{\text{community}}=q_{1}[-p_{1}C_{1}+(1-p_{1})(\mathrm{\tau F}-C_{1})]$$ (2)

By calculating the first-order partial derivative for domestic waste sorting probability ( $p_{1}$ ) and waste sorting supervision probability ( $q_{1}$ ) using $U_{\text{resident}}$ and $U_{\text{community}}$ , and setting the partial derivative to 0 based on the function maximization condition, the following relationship can be derived:


$$q^{*}=\frac{R_{2}-C_{2}}{\tau (R_{1}+F)}p^{*}=\frac{\mathrm{\tau F}-C_{1}}{\mathrm{\tau F}}$$ (3)

In the above Equation 3, $q^{*}$ and $p^{*}$ are solutions to the mixed strategy Nash equilibrium of the game between communities and residents. When the probability of domestic waste sorting supervision in the community exceeds $q^{*}$ , residents will sort domestic waste; otherwise, they will not. Similarly, if the probability of residents participating in domestic waste sorting is greater than $p^{*}$ , no regulatory actions will be taken by the community; otherwise, measures will be implemented. When beyond the Nash equilibrium solution, any strategy implemented by the communities or residents will have an identical effect. For $q^{*}$ , increased waste sorting supervision (*F*, τ , and $R_{1}$ ), along with higher benefits and lower costs associated with domestic waste sorting, results in higher probability of residents participating in domestic waste sorting. Therefore, the following research hypothesis H1 is proposed.

*Hypothesis H1*: The implementation of waste sorting supervision will cause an increase in residents' participation in domestic waste sorting.

It is worth clarifying that waste sorting supervision, as conceptualized in this study, differs from related constructs in the existing literature. Unlike subjective norms in the Theory of Planned Behavior, which refer to internally perceived social pressure from family or friends (17–19), supervision is an institutionalized, community-level enforcement mechanism involving designated personnel and formal rules. It also differs from macro-level policy pressure (27) in that it operates at the micro-level of actual waste disposal, and from general external constraints in that it encompasses not only deterrence but also information transmission, social norm reinforcement, and cognitive improvement (19, 23, 26–28). Through these complementary pathways, supervision functions as a multifaceted intervention rather than mere coercion.

The initial objective of waste sorting supervision is to enhance residents’ motivation to participate in domestic waste sorting. Increased supervision demonstrates a greater significance that the state places on domestic waste sorting, which, in turn, fosters residents’ awareness of its importance and their acknowledgement to its necessity, thus encouraging them to participate actively. When examining residents’ waste sorting behavior, it is critical to consider their knowledge of waste sorting practices (28). During the waste sorting supervision process, the primary role of community waste sorting supervisors is to educate and persuade residents who do not sort their domestic waste, guiding them into the proper waste sorting practices. The chief objective of waste sorting supervision is not punitive in nature. Instead, it involves the promotion and dissemination of sorting policies and standards to community residents, aiming to guide them to engage in proper practices. Furthermore, waste sorting supervision may compel residents to actively learn about policies and standards related to domestic waste sorting, thus enabling them to sort waste correctly. Based on that, the following research hypothesis H2 is proposed.

*Hypothesis H2*: The implementation of waste sorting supervision will increase residents' awareness on the significance, standards and policies of domestic waste sorting to motivate them into the practice.

## 3 Methodology

### 3.1 Empirical model

As domestic waste sorting behavior, namely, the explained variable, falls under binary choices, a binary Probit model is developed. The model benchmark form is established as follows:


$$\text{Sortin}g_{i}=\alpha_{0}+\alpha_{1}\text{Supervisio}n_{i}+\sum \gamma_{j}\text{Contro}l_{\mathrm{ij}}+\varepsilon_{i}$$ (4)

In Equation 4, $\text{Sortin}g_{i}$ represents the domestic waste sorting behavior of resident *i*, while $\text{Supervisio}n_{i}$ denotes the waste sorting supervision faced by resident *i*. In addition, $\text{Contro}l_{\mathrm{ij}}$ represents the *j*^th^ control variable of resident *i*, where $\alpha_{0}$ is a constant term, $\alpha_{1}$ and $\gamma_{j}$ are the parameters to be estimated, and $\varepsilon_{i}$ is the residual disturbance term.

To investigate the effect of waste sorting supervision on residents’ waste sorting behavior, this paper applies the methods of Baron and Kenny (31) and establishes a multiple mediating effect model as follows:


$$M_{i}=\delta_{0}+\delta_{1}\text{Superviso}n_{i}+\sum \beta_{j}\text{Contro}l_{\mathrm{ij}}+\tau_{i}$$ (5)

$$\text{Sortin}g_{i}=\varphi_{0}+\varphi_{1}\text{Supervisio}n_{i}+\varphi_{2}M_{i}+\sum \rho_{j}\text{Contro}l_{\mathrm{ij}}+\omega_{i}$$ (6)

In Equations 5, 6, $M_{i}$ is a mediating variable denoting the awareness of resident *i* on significance, standards and policies for waste sorting. $\delta_{0}$ and $\varphi_{0}$ are constant terms, whereas $\delta_{1}$ , $\varphi_{1}$ , $\beta_{j}$ , and $\rho_{j}$ are the parameters that require estimation. $\tau_{i}$ and $\omega_{i}$ are the residual disturbance terms.

### 3.2 Data

This paper utilizes data collected via a questionnaire survey conducted among urban residents in Beijing in 2022. Due to the restrictions caused by the COVID-19 pandemic, the study was conducted online using Wenjuanxing, a popular questionnaire platform in China. Prior to the official online survey, this study performed an initial offline pre-survey to gain insights into the waste sorting practices conducted by Beijing’s residents, and subsequently designed and refined the questionnaire accordingly. The study collected a total of 777 questionnaires from all districts in Beijing. After removing invalid responses, 720 valid samples were obtained for this study, the effective rate of the questionnaire reaching 92.7%. It should be noted that the COVID-19 pandemic may have influenced residents’ behavioral patterns, their trust in public policies, and the enforceability of community supervision during the survey period, which is further discussed in the concluding section. Additionally, as the survey was conducted online, the sample may be subject to selection bias—online respondents tend to be younger, more educated, and more digitally literate than the general population—which should be considered when generalizing our findings beyond Beijing’s urban core.

### 3.3 Variables and descriptive statistics

The explained variable in this paper pertains to residents’ participation in domestic waste sorting, where a binary value of 1 (participation) or 0 (non-participation) was assigned accordingly. As per the survey data, 591 residents, comprising 82.1% of the full sample, participated in domestic waste sorting, whereas the remaining 17.9% of residents did not sort their domestic waste. Moreover, during the period of the COVID-19 pandemic, the phenomenon of littering increased (23).

The core explanatory variable in this paper is waste sorting supervision, measured through a questionnaire item that inquires whether or not the respondent’s community implements strict supervision of waste sorting practices. The specific question asked is “Is your community strict in waste sorting supervision?.” Responses were on a 5-point Likert-type scale ranging from 1 (extremely loose) to 5 (extremely strict). A total of 210 residents, accounting for 29.2% of the entire sample, answered with “relatively strict” or “very strict.” This indicates that the overall waste sorting supervision in the communities is not stringent enough, as residents tend to dispose of their garbage randomly, and supervisors are unable to continuously supervise the process.

This paper proposes the inclusion of three mediating variables, namely, (i) Awareness of sorting significance: the questionnaire item is measured by asking “Do you think domestic waste sorting is necessary?,” and responses were on a 5-point Likert-type scale ranging from 1 (extremely unnecessary) to 5 (extremely necessary); (ii) Awareness of sorting standards: the questionnaire item is measured by asking “Are you aware of sorting standards for domestic waste?,” and responses were on a 5-point Likert-type scale ranging from 1 (extremely unfamiliar) to 5 (extremely familiar); and (iii) Awareness of sorting policies: the questionnaire item is measured by asking “Are you aware of sorting policies for domestic waste?,” and responses were on a 5-point Likert-type scale ranging from 1 (extremely unfamiliar) to 5 (extremely familiar).

With reference to existing studies (23, 32–34), variables including residents’ age, gender, education, occupation, family size, family income, dietary habits, distance from waste drop-off point, residential years, residential environment, and sense of community are introduced to address the potential impact of control variables on domestic waste sorting. The details of each variable are shown in Table 1.

**Table 1:** Variable definition and descriptive statistics.

Variables	Definition	Mean	SD
Dependent variable			
Domestic waste sorting	sorting = 1, non-sorting = 0	0.821	0.384
Core explanatory variable			
Waste sorting supervision	The degree of community waste sorting supervision, from very loose to very strict, is assigned from 1 to 5 in turn.	2.871	1.089
Mediating variables			
Awareness of sorting significance	Do you think domestic waste sorting is necessary? The scale ranges from extremely unnecessary to extremely necessary, assigned from 1 to 5 in turn.	4.386	0.888
Awareness of sorting standards	Are you aware of sorting standards for domestic waste? The scale ranges from completely unaware to completely aware, assigned from 1 to 5 in turn.	3.838	0.762
Awareness of sorting policies	Are you aware of sorting policies for domestic waste? The scale ranges from completely unaware to completely aware, assigned from 1 to 5 in turn.	3.535	0.890
Control variables			
Gender	Male = 1, female = 0	0.381	0.486
Age	Respondent’ age (years)	31.960	8.481
Education	High school and below = 1, junior college = 2, undergraduate = 3, and graduate and above = 4.	3.011	0.719
Occupation	Student = 1, doctor = 2, civil servant = 3, teacher = 4, and others = 5.	3.904	1.435
Family size	Number of household members (persons)	3.349	1.267
Family income	1 for less than 50,000 yuan, 2 for 50,000–100,000 yuan, 3 for 100,000–150,000 yuan, 4 for 150,000–200,000 yuan, 5 for 200,000–250,000 yuan, and 6 for 250,000 yuan and above.	4.192	1.508
Dietary habits	How often do you prepare and consume meals at home? The scale ranges from almost never to almost everyday, assigned from 1 to 5 in turn.	4.003	1.043
Distance from waste drop-off point	Distance within 5 min = 0, distance of 5 min or more = 1.	0.938	0.242
Residential years	Years of residence in the community (years)	5.175	2.555
Residential environment	New community = 1, old community = 0	0.713	0.453
Sense of community	A 5-point Likert scale from very weak to very strong	3.625	0.886

## 4 Results and discussion

### 4.1 Basic regression analysis

Referring to Zhai et al. (35), Probit model parameters and marginal effects of each explanatory variable are presented in Table 2. The results show that waste sorting supervision has a significant positive effect on urban residents’ domestic waste sorting, regardless of the consideration of control variables. We further changed the waste sorting supervision variable into a binary variable, and the estimation results remained consistent (see Table A1). This implies that stricter supervision of waste sorting in the communities is associated with a higher likelihood of residents’ active engagement in domestic waste sorting. These findings are consistent with previous studies (1, 16), and validates hypothesis H1. The reason for this may be attributed to the external constraint imposed by domestic waste sorting supervision on residents. Failure to sort domestic waste could lead to reminders, criticism or education by community waste sorting supervisors when residents deposited their waste at the district collection point. The experience of external interventions may sensibly stimulate them to engage in domestic waste sorting. In communities where domestic waste sorting supervision was more stringent, some residents could be subject to punishment for failing to sort waste. Confronted with this potential outcome, these residents may participate in domestic waste sorting to avoid such penalties. Previous research has suggested that people tend to be highly responsive to punishment measures, highlighting the need for effective policies to successfully implement such measures (23). And punishment measures have been found to have the most significant impact on residents’ waste sorting behavior (25). Thus, there is a need to implement supervision measures to encourage residents’ participation in domestic waste sorting, particularly during the early stages of the waste sorting policy’s implementation (1).

**Table 2:** Basic regression results.

Variables	Probit model		Probit model (marginal effect)	
Core explanatory variable				
Waste sorting supervision	0.579*** (0.054)	0.538*** (0.070)	0.127*** (0.011)	0.102*** (0.012)
Control variables				
Gender		−0.024 (0.136)		−0.005 (0.026)
Age		0.039*** (0.010)		0.007*** (0.002)
Education (with “high school and below “as the reference group)				
Junior college		0.300 (0.352)		0.050 (0.060)
Undergraduate		−0.025 (0.274)		−0.005 (0.051)
Graduate and above		−0.180 (0.307)		−0.036 (0.059)
Occupation (with “student” as the reference group)				
Doctor		0.143 (0.447)		0.027 (0.083)
Civil servant		−0.071 (0.237)		−0.015 (0.048)
Teacher		0.480 (0.306)		0.081 (0.051)
Others		0.061 (0.220)		0.012 (0.044)
Family size		0.072 (0.053)		0.014 (0.010)
Family income		0.170*** (0.046)		0.032*** (0.009)
Dietary habits		0.123** (0.061)		0.023** (0.011)
Distance from waste drop-off point		0.023 (0.252)		0.004 (0.048)
Residential years		0.008 (0.028)		0.002 (0.005)
Residential environment		0.028 (0.148)		0.005 (0.028)
Sense of community		0.131* (0.076)		0.025* (0.015)
Constant	−0.568*** (0.148)	−3.566*** (0.565)		
Rseudo *R*^2^	0.156	0.272		
Wald chi^2^	113.31	147.45		
Number of obs	720	720	720	720

The values in parentheses are robust standard errors. ***p < 0.01, **p < 0.05, *p < 0.1.

Regarding the control variables, the age of the respondents is statistically significant at the 1% level, with a positive correlation indicating that older residents are more inclined towards participating in domestic waste sorting. This result is consistent with the study of Kuang and Lin (36). Total household income was found to have a positive and statistically significant effect on residents’ participation in domestic waste sorting. The study revealed that residents with higher household incomes are more likely to be motivated to participate in domestic waste sorting. One possible explanation for this outcome is that residents with higher incomes typically had better access to waste sorting information, compared to lower-income ones (33). Dietary habits were also found to significantly influence residents’ behavior towards domestic waste sorting. The study discovered that residents with a higher frequency of cooking at home demonstrated a greater inclination towards sorting domestic waste, particularly kitchen waste, which is more commonly generated during home cooking (37). Furthermore, individuals who frequently dropped off their waste at community waste collection points were more likely to be supervised, leading to an increased probability of being penalized for not sorting waste. Residents’ sense of community had a significant impact on their behavior towards domestic waste sorting. The study indicates that residents with a strong sense of community were more likely to engage in activities that would benefit the community and adhere to its regulations, such as domestic waste sorting.

### 4.2 Endogeneity test

To address potential endogeneity concerns, this study employs whether a resident’s residential community is under gated management as an instrumental variable for waste sorting behavior. This variable is measured by the survey question, “Is your residential community under gated management?” with responses assigned as 1 for “yes” and 0 for “no.” Theoretically, whether a community is under gated management satisfies both the relevance and exogeneity conditions required for a valid instrumental variable. On the one hand, gated management is typically accompanied by a more comprehensive property management system and clearer community governance responsibilities, which facilitate the uniform establishment of waste sorting drop-off points, the implementation of door-to-door publicity campaigns, and the enforcement of designated disposal times—thereby significantly enhancing the convenience and standardization of residents’ participation in waste sorting. Consequently, there exists a strong correlation between gated management and residents’ waste sorting behavior, fulfilling the relevance condition. On the other hand, gated management essentially reflects differences in community governance models rather than directly intervening in residents’ individual attitudes or willingness toward waste sorting. It provides external institutional support for waste sorting behavior without directly altering residents’ intrinsic motivations or daily habits. Therefore, this variable does not influence residents’ participation in waste sorting through channels other than waste sorting behavior itself, thereby satisfying the exogeneity condition.

Given that both residents’ waste sorting behavior and waste sorting regulation are discrete variables, this study employs the Conditional Mixed Process (CMP) approach proposed by Roodman (38) to test for endogeneity. The results are presented in Table 3. The first-stage regression results show that the instrumental variable is statistically significant at the 1% level, indicating a strong correlation between the instrumental variable and waste sorting regulation. The second-stage regression results reveal that, after accounting for endogeneity, the effect of waste sorting regulation on urban residents’ waste sorting behavior remains significantly positive, further supporting the conclusion that waste sorting regulation promotes residents’ participation in waste sorting. Additionally, the endogeneity test parameter (atanhrho_12) fails to reject the null hypothesis that waste sorting regulation is exogenous, suggesting that the benchmark analysis does not suffer from severe endogeneity issues and that the model estimates are robust.

**Table 3:** Estimation results of endogeneity test.

Variables	CMP method	
	First stage	Second stage
Core explanatory variable		
Waste sorting supervision		0.950*** (0.211)
Whether residential community under gated management	0.394*** (0.069)	
Control variables	Controlled	Controlled
atanhrho_12	−0.455 (0.292)	
Number of obs	720	720

The values in parentheses are robust standard errors. ***p < 0.01.

However, we acknowledge that the exclusion restriction may not be fully satisfied. Gated communities often differ from non-gated ones in socioeconomic composition, property management quality, resident homogeneity, and pre-existing environmental norms, all of which could potentially influence residents’ waste sorting behavior through channels other than supervision. To assess this concern, we conduct a reduced-form regression of gated management on waste sorting behavior (without controlling for supervision). As shown in Table A2, the estimated coefficient is 0.362 (*p* < 0.01), indicating that gated management has a significant direct association with sorting behavior. This suggests that our IV estimate should be interpreted with caution, as part of the total effect may operate through unobserved community-level characteristics rather than supervision alone. While we have controlled for observable community and individual characteristics in our main regressions, we acknowledge the possibility that unmeasured confounders could violate the exclusion restriction. This limitation is further discussed in the concluding section.

### 4.3 Heterogeneity analysis

To facilitate further research into the heterogeneity of the effect of waste sorting supervision on urban residents’ behavior towards waste sorting, this paper categorizes the sample into different groups based on age and income: low- and high-age groups, and low- and high-income groups, respectively. With reference to the studies by Zhai et al. (39), age was determined based on the mean age of the entire sample, and those above this mean were classified as the high age group, while those below it were classified into the low age group. For income, a threshold of 150,000 yuan was established, and households with a pre-tax income of 150,000 yuan or less were categorized as the low-income group, while those with a pre-tax income exceeding 150,000 yuan were designated as the high-income group. The Probit model regression estimation was conducted separately for the four-component samples, and the obtained results are presented in Table 4.

**Table 4:** Results of heterogeneity analysis.

Variables	Grouped by age (marginal effect)		Grouped by income (marginal effect)	
	Low age	High age	Low income	High income
Core explanatory variable				
Waste sorting supervision	0.137*** (0.019)	0.067*** (0.016)	0.103*** (0.023)	0.092*** (0.014)
Control variables	Controlled	Controlled	Controlled	Controlled
Rseudo *R*^2^	0.253	0.249	0.285	0.250
Wald chi^2^	89.56	59.83	63.64	91.49
Number of obs	384	334	236	484

The values in parentheses are robust standard errors. ***p < 0.01.

Regarding age groups, waste sorting supervision demonstrated a statistically significant positive impact, at the 1% level, on the participation in domestic waste sorting for both low and high age groups. From the perspective of marginal effect, the study reveals that young residents facing stringent waste sorting supervision exhibited a 13.7% higher probability of participating in domestic waste sorting, as compared to the group facing lenient supervision. Additionally, old residents facing strict supervision of waste sorting demonstrated a 6.7% higher probability of participating in waste sorting activities, as compared to the group facing loose supervision. These results suggest that young residents were more inclined to participate in domestic waste sorting when faced with strict supervision, indicating that waste sorting supervision has a stronger impact on young residents’ engagement in the activities. Possible explanations for this trend include the fact that younger residents, particularly students, tended to be more receptive to various policies and regulations. And waste sorting is a pro-environmental behavior and previous studies have shown that younger individuals tended to hold a greater concern for the environment (40).

Regarding income groups, the results of the study indicate that waste sorting supervision had a statistically significant and positive effect, at the 1% level, on the participation in domestic waste sorting by both low- and high-income residents. From the perspective of marginal effect, the study reveals that low-income residents facing stringent waste sorting supervision exhibited a 10.3% higher probability of participating in domestic waste sorting, as compared to the group facing lenient supervision. Additionally, high-income residents facing strict supervision of waste sorting demonstrated a 9.2% higher probability of participating in domestic waste sorting, as compared to the group facing loose supervision. These results suggest that low-income residents were more inclined to participate in domestic waste sorting when faced with strict supervision, indicating that waste sorting supervision had a stronger effect on low-income residents’ engagement in the activities. One possible explanation is that low-income residents tended to be more risk-averse (41, 42), and had heightened concerns about the potential consequences of non-participation in waste sorting activities, including punishments and fines. This finding is of particular significance as it indirectly supports the feasibility of implementing domestic waste sorting in rural areas (43). Despite having a lower income level on average compared to their urban counterparts, rural residents were not necessarily less motivated to engage in domestic waste separation activities.

### 4.4 Robustness test

To enhance the robustness of the above empirical findings, this study conducts a series of robustness tests by substituting the explained variable in the regression analysis. To evaluate residents’ waste sorting behavior, the duration of their involvement is utilized. As duration is a limited dependent variable, taking on values greater than or equal to zero, this paper employs the Tobit model for estimation. Table 5 displays the estimated outcomes for both the full sample and the sub-sample. The supervisions of waste sorting are both significantly positive at the statistical level of 1%. As indicated by the results, waste sorting supervision had a stronger positive effect on the participation of low-age and low-income residents in waste sorting than on their counterparts. These findings align consistently with the outcomes of the analysis presented above, thus confirming the robustness of the results obtained in this study.

**Table 5:** Robustness test results.

Variables	Full sample (marginal effect)	Grouped by age (marginal effect)		Grouped by income (marginal effect)	
		Low age	High age	Low income	High income
Core explanatory variable					
Waste sorting supervision	0.467*** (0.073)	0.626*** (0.108)	0.313*** (0.097)	0.484*** (0.145)	0.434*** (0.084)
Control variables	Controlled	Controlled	Controlled	Controlled	Controlled
Rseudo *R*^2^	0.082	0.089	0.068	0.077	0.076
LR chi^2^	228.76	128.68	88.27	66.53	143.52
Number of obs	720	384	336	236	484

The values in parentheses are robust standard errors. ***p < 0.01.

### 4.5 Mechanism analysis

In this paper, the mediating effect model is further used to explore the action path of waste sorting supervision in promoting residents’ participation from three aspects: residents’ awareness on sorting significance, standards and policies. Given that awareness on these aspects entails an ordinal variable, the paper employs the Ordered Probit model to carry out regression and estimate the marginal effect of each explanatory variable. The results are shown in Table 6.

**Table 6:** Results of mechanism analysis.

Variables	Awareness of sorting significance	Waste sorting	Awareness of sorting standards	Waste sorting	Awareness of sorting policies	Waste sorting
Core explanatory variable						
Waste sorting supervision	0.072*** (0.018)	0.093*** (0.012)	0.062*** (0.010)	0.080*** (0.012)	0.061*** (0.009)	0.085*** (0.012)
Awareness of sorting significance		0.055*** (0.012)				
Awareness of sorting standards				0.114*** (0.016)		
Awareness of sorting policies						0.077*** (0.014)
Control variables	Controlled	Controlled	Controlled	Controlled	Controlled	Controlled
Constant		−4.678*** (0.617)		−5.630*** (0.709)		−4.739*** (0.627)
Rseudo *R*^2^	0.073	0.300	0.103	0.356	0.109	0.314
Wald chi^2^	120.70	172.83	139.40	157.30	175.18	168.84
Number of obs	720	720	720	720	720	720

The values in parentheses are robust standard errors. ***p < 0.01.

Regarding residents’ awareness on the significance of domestic waste sorting, the results indicate that the supervision had a notably positive effect on this aspect. The findings highlight the role of supervision in increasing residents’ awareness. Furthermore, the findings suggest that awareness on domestic waste sorting was a significantly positive at a statistical level of 1%. This outcome indicates that there existed a partial mediating effect of such awareness between the supervision of domestic waste sorting and residents’ waste sorting behavior, thereby confirming hypothesis H2. Waste sorting supervision strategy changes residents’ perception, such as the importance and the meaning of waste sorting (26). The implementation of waste sorting supervision highlights the growing attention that the state places on domestic waste disposal as well as the significance of household waste sorting. As a result, when residents develop a better awareness on the significance of domestic waste sorting, they are more likely to actively participate in the endeavour.

The study finds that domestic waste sorting supervision has a significant positive effect on improving residents’ awareness of waste sorting standards at the 1% level of significance. Thus, strengthening supervision efforts can enhance residents’ perception and adherence to domestic waste sorting standards. The analysis revealed that both the perception of waste sorting standards and the supervision of domestic waste sorting, after both being included into the model, had a statistically significant positive effect at the 1% level. These findings signify that there existed a partial mediating effect of standard perception between waste sorting supervision and residents’ domestic waste sorting behavior, thereby confirming hypothesis H2. Policy instruments such as waste sorting supervision are beneficial for the public to gain knowledge about waste sorting (27). Furthermore, community waste sorting supervisors played a role in identifying and correcting inappropriate domestic waste sorting and disposal practices by residents. They could provide reminders and educational interventions to ensure correct practices.

Regarding residents’ awareness on the policies for domestic waste sorting, the analysis indicates that the effect of waste sorting supervision on their policy perception is significantly positive at the 1% level, and that strengthening the supervision can improve residents’ policy perception. The analysis reveals that both the awareness of waste sorting policies and the supervision of domestic waste sorting, after both being included into the model, had a statistically significant positive effect at the 1% level. These findings signify that there existed a partial mediating effect of policy perception between waste sorting supervision and residents’ sorting behavior, thus confirming hypothesis H2. The process of domestic waste sorting supervision also involves the dissemination of polices on domestic waste sorting, through which residents are able to better understand and become familiar with these policies. Policy effectiveness is helpful in shaping households’ pro-environmental behaviors by altering residents’ attitudes (32).

## 5 Conclusion

Domestic waste sorting is the embodiment of social civilization and progress, reflecting an inevitable trend in global development. Some countries have gradually transitioned from encouraging residents to participate in domestic waste sorting to imposing penalties for non-compliance. Failure to sort domestic waste will face varying degrees of punishment. This paper uses survey data collected from urban residents in Beijing to empirically examine the impact of waste sorting supervision on urban residents’ domestic waste sorting. The results show that stricter waste sorting supervision was associated with a higher probability of residents’ engagement in domestic waste sorting. In addition, residents’ age, household income, dietary habits, and sense of community were significant determinants affecting their participation in domestic waste sorting. The sub-sample regression results show that the effect of supervision on residents’ domestic waste sorting displayed a marked heterogeneity in age and income, with a more pronounced impact found among low-income and young residents relative to their high-income and older counterparts. The mechanism analysis suggests that waste sorting supervision may enhance residents’ participation in domestic waste sorting by raising their awareness of waste sorting significance, standards, and policies.

Based on the above findings, the following policy implications can be derived: currently, there remains significant potential for enhancing urban residents’ participation in domestic waste sorting, and effective waste sorting supervision and management are important necessities. This is particularly relevant in light of the COVID-19 pandemic, which has had an impact on the formation of positive waste management behaviors. For instance, without the support and guidance of volunteers, there has been an increase in littering (23). Therefore, strengthening supervision of waste sorting, along with reminders and education to encourage active participation, may be an important consideration for policymakers. Effective supervision of domestic waste sorting should focus not only on punitive measures but also on incorporating public education and awareness campaigns into its implementation. Through promoting and disseminating information on the practical significance of domestic waste sorting, its related policies and standards to community members—particularly low-income and younger residents—meaningful progress can be achieved in fostering a conscientious waste sorting culture. Such efforts are vital to creating healthy, clean, and sustainable communities. Importantly, the effectiveness of front-end supervision ultimately depends on the integrity of the entire waste management chain. Mixed collection and transportation, a persistent problem in some Chinese cities, can severely undermine residents’ trust in waste sorting policies and offset the gains from front-end supervision. Therefore, strengthening supervision of residents’ sorting behavior should be accompanied by parallel efforts to improve downstream infrastructure, including the separation of collection and transportation systems and the development of recycling and resource recovery facilities. Only when front-end sorting is matched by back-end treatment can supervision achieve its full potential in fostering sustainable waste management practices.

This study also has several limitations. First, due to questionnaire constraints, key parameters in our game-theoretic model, such as fine amounts, detection probability, sorting costs, and benefits, were not directly measured. Second, our measure of waste sorting supervision relies on a single-item subjective evaluation of perceived strictness, which may not fully capture the multi-dimensional nature of supervision, and may be subject to common method bias and reverse causality concerns. Third, our data were collected during the COVID-19 pandemic, which may have altered residents’ behavioral patterns, their trust in public policies, and the enforceability of community supervision, yet we were unable to incorporate these pandemic-related factors into our empirical analysis. Fourth, the instrumental variable used to address endogeneity may not fully satisfy the exclusion restriction, as gated communities may differ in socioeconomic composition, property management quality, and pre-existing environmental norms, all of which could affect sorting behavior independently of supervision. Fifth, our measure of waste sorting behavior is self-reported and thus susceptible to social desirability bias, which may overestimate actual participation rates; we did not validate self-reports against objective observations, and future research could incorporate field audits or sensor-based monitoring to mitigate this bias. Sixth, we were unable to control for residents’ housing tenure status, which may be relevant in the Beijing context as homeowners might exhibit stronger community commitment and thus higher sorting compliance than renters; this omission may affect the precision of our estimates. All in all, future research could design more comprehensive survey instruments or field experiments to operationalize the structural parameters of the model, capture the multi-dimensionality of supervision, employ alternative IVs or quasi-experimental designs to strengthen causal identification, and examine potential pandemic-related moderating effects.

## Data Availability

The raw data supporting the conclusions of this article will be made available by the authors, without undue reservation.

## Appendix

**Table A1:** Robustness test results (1).

Variables	Probit model (marginal effect)		
Core explanatory variable			
Waste sorting supervision	0.166*** (0.032)	0.151*** (0.025)	0.303*** (0.061)
Control variables	Controlled	Controlled	Controlled
Rseudo *R*^2^	0.222	0.236	0.251
Wald chi^2^	123.04	144.78	116.50
Number of obs	720	720	720

The values in parentheses are robust standard errors. ****p* < 0.01. In column (2), the response option “extremely loose” for waste sorting supervision is assigned as 0, and all other responses are assigned as 1. In column (3), the response options “extremely loose” and “relatively loose” are assigned as 0, and all other responses are assigned as 1. In column (4), the response options “extremely loose,” “relatively loose” and “moderate” are assigned as 0, and all other responses are assigned as 1.

**Table A2:** Impact of gated management on domestic waste sorting.

Variables	Probit model		Probit model (marginal effect)	
Core explanatory variable				
Whether residential community under gated management	0.469*** (0.113)	0.362*** (0.128)	0.119*** (0.028)	0.076*** (0.026)
Control variables	Uncontrolled	Controlled	Uncontrolled	Controlled
Rseudo *R*^2^	0.026	0.198		
Wald chi^2^	17.18	116.25		
Number of obs	720	720	720	720

The values in parentheses are robust standard errors. ****p* < 0.01.
